# An inquiry into good hospital governance: A New Zealand-Czech comparison

**DOI:** 10.1186/1478-4505-4-2

**Published:** 2006-02-06

**Authors:** Elizabeth Ditzel, Pavel Štrach, Petr Pirozek

**Affiliations:** 1 Department of Management, University of Otago, School of Business, P.O.Box 56, Dunedin, New Zealand; 2 Department of Business Management, University of Economics, Prague, Faculty of Management, Jarosovska 1117/II., 377 01 Jindrichuv Hradec, Czech Republic

## Abstract

**Background:**

This paper contributes to research in health systems literature by examining the role of health boards in hospital governance. Health care ranks among the largest public sectors in OECD countries. Efficient governance of hospitals requires the responsible and effective use of funds, professional management and competent governing structures. In this study hospital governance practice in two health care systems – Czech Republic and New Zealand – is compared and contrasted. These countries were chosen as both, even though they are geographically distant, have a universal right to 'free' health care provided by the state and each has experienced periods of political change and ensuing economic restructuring. Ongoing change has provided the impetus for policy reform in their public hospital governance systems.

**Methods:**

Two comparative case studies are presented. They define key similarities and differences between the two countries' health care systems. Each public hospital governance system is critically analysed and discussed in light of D W Taylor's nine principles of 'good governance'.

**Results:**

While some similarities were found to exist, the key difference between the two countries is that while many forms of 'ad hoc' hospital governance exist in Czech hospitals, public hospitals in New Zealand are governed in a 'collegiate' way by elected District Health Boards. These findings are discussed in relation to each of the suggested nine principles utilized by Taylor.

**Conclusion:**

This comparative case analysis demonstrates that although the New Zealand and Czech Republic health systems appear to show a large degree of convergence, their approaches to public hospital governance differ on several counts. Some of the principles of 'good governance' existed in the Czech hospitals and many were practiced in New Zealand. It would appear that the governance styles have evolved from particular historical circumstances to meet each country's specific requirements. Whether or not current practice could be improved by paying closer attention to theoretical models of 'good governance' is debatable.

## Background

A generally accepted definition of governance stipulates the responsibility and accountability for the overall organisation of the operation of an organisation. More specifically, hospital governance has been conceived of as a shared process of top-level organisational leadership, policy making and decision making [1]. The governance process is orchestrated by boards – a group of people who are ideally charged with responsibility and accountability for the overall performance of the organisation. However, in a recent comprehensive review of health care governance, and with specific reference to OECD countries such as Canada that began experimenting with the governance function of health delivery organisations in the late 1990s, it was concluded that "new governance models have appeared lately that defy the first principles of good governance" [[2]:108]. D W Taylor [2] also proposed that nine principles of 'good governance' could be applied to health care management to combat this trend.

Given the importance of the governance function in managing hospitals, here it is suggested that it may be timely for policy makers and citizens alike of countries experiencing health sector change such as New Zealand and the Czech Republic, to first consider to what extent their current models of hospital governance meet the specified 'good governance' criteria, and second to address the implications of 'good governance' for their respective country's health care environments.

While it is clear at the outset that as countries New Zealand and the Czech Republic are quite different in nature, historical circumstances, economic performance and geographical location, several similarities between the two countries' health care systems are apparent at a macro level of analysis. Both countries have plural health care systems with a declared universal right to free hospital care and a reputation for delivering high quality health care in their respective regions [3-5] and each has experimented with greater competition among hospitals and with enforcement of their efficiency, quality and responsiveness [6,7]. However, the resulting public hospital governing structures are quite different with a more formalized 'collegiate' system being in place in New Zealand, and an apparent 'ad hoc' system operating in the Czech Republic.

Throughout the paper New Zealand is used as a case study of a care system that has undergone, survived and learned from several major transitions. Change was primarily driven by a period of radical economic reform – colloquially referred to as 'Rogernomics' – beginning in the 1980s and continuing in less dramatic fashion into the next two decades [8]. It is compared with the Czech Republic which as a state under Soviet influence (until 1989) practiced a centrally planned forcedly egalitarian economy until the Czech Velvet Revolution liberalised its economic and political systems. Although New Zealand now has an economic system which internationally is considered to have many successful attributes [7-10], like most other OECD countries such as the Czech Republic, it has struggled to establish the best way of organising and delivering publicly financed health care services. Thus, it is argued that the two countries have in common a history of recent major structural economic reform which has had a major impact on the development and performance of their health care systems [3,4,11]. The mutual experience of ongoing institutional restructuring and reform fit the pattern reported in recent comparative health services literature suggesting that "the most remarkable feature of health care system reform among the 17 [OECD] countries is the degree of emerging convergence...in the general direction of those pioneered in other countries" [[12], 5:45].

First, this paper presents a descriptive analysis of the two countries as case studies of health care sector reform. This provides a comparative overview of the internal structural elements of each country's public hospital governance system and demonstrates how hospitals as health care institutions fit into the external health care system framework. Second, hospital governance practice in each country is discussed in light of D W Taylor's principles of 'good governance' [2]. Finally, conclusions and recommendations for future research are presented.

## Methods

A literature review was conducted to collect relevant descriptive and statistical data that is contained in Table 1. Then a comparative case study methodology [13] was used to gain information to empirically investigate whether or not D W Taylor's [2] principles of good governance were applied in the New Zealand and Czech Republic health care contexts. This investigative approach was taken due to a lack of an accepted taxonomy in health care systems' research that would provide detailed information on eligibility, benefits, reimbursement, financing and delivery of health care services [14]. Another difficulty in this research area is the dearth of available literature reporting economic and health outcome performance data that is easily comparable.

**Table 1:** Comparison of health care indicators

Indicator	Czech Republic	New Zealand
**Public health care coverage, per cent of population**	100	100
**Life expectancy at birth in years (2000)**		
**Males**	71.7	75.7
**Females**	78.4	80.8
**GDP per capita (PPP, USD, 2003)**	15,700	21,600
**Health expenditure as a percentage of GDP (2002)**	7.4	8.5
**Public expenditure on health as a per cent of trend GDP (2000)**	6.5	6.2
**Health expenditure per capita (PPP, USD, 2002)**	1,118	1,857
**Infant mortality (Deaths per 1,000 live births, 2000)**	4.1	5.8
**Practicing physicians per 1,000 population (2002)**	3.5	2.1
**Practicing nurses per 1,000 population (2002)**	9.4	9.4
**Discharges (all causes) per 100,000 population (2002)**	21,861	20,555

This preliminary part of the literature review process is derived from an assessment of relevant publications including government reports, working papers and academic articles concerning the two health care systems.

Table 1 presents a summary comparison of key health care indicators of the two country's health care and hospital systems [5,6,11,15-21] thereby establishing the credentials for further analysis. It demonstrates that there are close similarities between the two countries in several key macro economic health indicators, e.g. health expenditure as a percentage of GDP and per capita, the number of practising medical staff (nurses and doctors), and the number discharged from hospital. This information provides the reader with an outline of the contextual background for the paper.

## Results

### Case 1 – New Zealand

New Zealand was the first country to create a universal social security system and free health in the 1930s. This reflects a strong socialist tradition and it was the socialist (Labour) party that introduced deregulation and market reforms in the 1980s. These changes attracted much international attention and New Zealand has often been reported as a "success story across the globe" [[8]: vii] in structural economic reforms. Changes to the health sector were founded on the idea that competition between providers would deliver the improved technical facilities and cost efficiency, while competition between purchasers would have improved allocative efficiency by making them more responsible for their own health. However, by 1996, the competitive system was abandoned. It was changed back to a co-operative system in which the roles of purchaser and provider of services to contractual arrangements are integrated and to a system where decisions are made locally by elected health boards.

**Figure 1:**
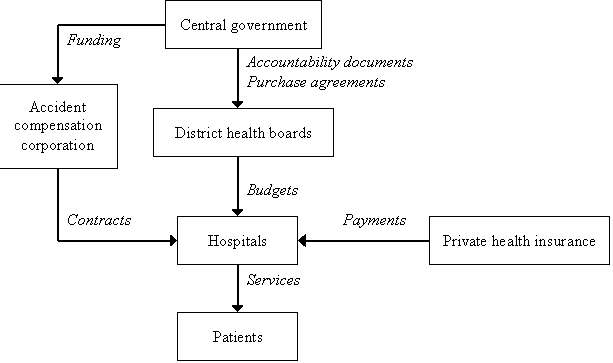
Structure of New Zealand health care system.

In 1999, a new Labour-led coalition government was elected. The government opposed the market model, arguing that it promoted unhealthy competitive tendering for contracts leading to fragmented services, lacked democratic community input, and was neither accountable to central government nor to local communities even though it had not been in place very long [10,4]. Shortly afterwards, the current structure of 21 District Health Boards (DHBs) was established. Like the previous area health boards, these boards are governed by a mix of locally elected and centrally appointed representatives and funded on a population basis. They hold the budget for secondary and primary health services and must provide these services themselves or purchase them from non-government providers. Figure 1 demonstrates how the New Zealand health care system currently operates [18,5].

### New Zealand public hospital governance

Widespread change in the health care sector brought with it a series of changes to the philosophical underpinnings and governance of public hospitals. The 'pro market' rationale of the 1980s emphasised the development of competition among the 14 locally elected area health boards created in 1989 that both funded and provided hospitals and some other services in their regions. However, reports about this managerialist system pointed to inefficiencies, poor management, budget overruns, lengthening waiting lists and badly eroded assets in the public hospitals [9,22-25]. In 1991 these health boards were seen as inadequate by a new conservative (National) government that responded by introducing market type reforms in which separate organisations were responsible for the purchasing and provision of health related services [26]. Hospitals became publicly owned companies called Crown Health Enterprises (CHEs) administered by boards of directors appointed by the Minister of Health based on their management skills. The CHEs were subject to normal company law and were required to earn a rate of return on capital comparable to that of the business sector. However, this competitive model did not deliver the intended results either. Many CHEs inherited and continued to report deficits, barriers to entry limited contestability, purchasers and providers of services struggled to establish contractual relationships. Transition costs were high and the expected savings were not made. While this experimental quasi market model provided some tangible benefits – average length of stay decreased, units costs fell, better information management systems facilitated greater accountability and better management of capital – the evidence suggested that the overall goal of greater efficiency was not achieved [10].

The next change came about in 1996 when New Zealand's first proportionally elected coalition government renamed the CHEs as 'Hospital and Health Services' (HSSs) and removed their 'for profit' status. Later, in 2000, 21 locally elected health boards were reinstated and New Zealand returned to a service model where boards plan most health and disability support and are responsible for the level, mix, and quality of the services and for meeting the health goals, targets and standards set by the Minister of Health (MoH) [26,27].

Today, the majority of New Zealand hospitals are either state owned and funded, or privately owned and partially funded by the state. Public hospitals are divided into geographical areas called DHBs. Funding for DHBs is provided by central government and is population-based, i.e., it is done on the basis of the particular requirements of the people living in the geographical location. The basic aim is to maximise the health benefit from available funds and to set priorities for demand within specified services [4,27]. The primary key performance objective of each DHB is to attain a fair and functional health care system that is effective in contributing to the health of New Zealanders [18].

Although all citizens are entitled to receive state funded free medical and surgical hospital care, those who choose to go to a private hospital and who do not have medical insurance must pay for services they receive. Private hospitals are owned and managed either by private medical insurance companies, individual investors or public charities. The Accident Compensation Corporation (ACC) provides universal cover for work-related illness and accidents for all New Zealand citizens. Private health insurance can be purchased by individuals for their own personal medical and/or surgical procedure cover or it may sometimes be provided by employers as a workplace benefit. Funds for ACC are collected from employers and employees by way of subsidies on wages and they are administered and distributed by central government to health care providers including hospitals [27].

### Case 2 – the Czech Republic

In Czechoslovakia in the late 1940s a universal national health service was created. It was largely free and funded predominantly through taxation. The system was administered locally as well as on a regional and district basis. Most acute inpatient hospital services were provided by different types of hospitals at a district level. Type 1 hospitals comprised about 250 beds and provided hospital care in general medicine, general surgery, paediatrics, obstetrics and gynaecology. Type II hospitals were larger (about 680 beds) and provided more specialities. Both types of hospitals often had attached clinics and outpatients departments. Type III hospitals were typically found at a regional level and provided more specialised services including tertiary referrals. In addition to these acute hospital services, non-acute beds were provided for 'spa' (rehabilitative) treatments and in mental hospitals [28].

**Figure 2:**
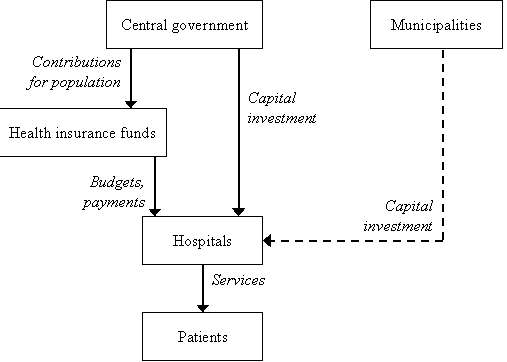
Structure of Czech health care system.

In 1993, Czechoslovakia split into the Czech Republic and Slovakia. Regions were abolished and hospitals were managed centrally, though local authorities were formally in charge. A publicly funded General Health Insurance Office was established and independent health providers, for instance hospitals and community physicians have contracts with this office. Some independent health insurance companies were also set up in order to encourage competition [29]. As was the case in New Zealand, these changes, which rejected public ownership and operation of the entire system, resulted from a perception of inefficiency in the systems in place during the 1980s and earlier times [3,16]. Figure 2 outlines the way the Czech Republic health care system currently operates (source abridged from [5]).

### Czech public hospital governance

The majority of Czech Republic hospitals are owned and operated by the state and municipal government. Military, state, and municipal hospitals are non-for-profit institutions; while private hospitals operate on profit-based principles. There are no private not-for-profit owned hospitals. Public hospitals are mainly university clinics and they are highly specialized facilities. State and military hospitals usually have simple organisational structures with a principal manager (called a Director), who reports to and is controlled by a given state department [3,21]. Such hospitals have neither a board of directors nor a supervisory board. Privately owned hospitals are divided between basic health care or on narrowly defined specialty areas such as cosmetic surgery. These are governed under standard business principles and focus on issues of effectiveness and profitability [30-32].

**Figure 3:**
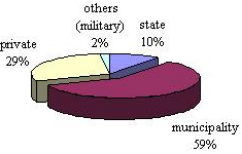
'Ownership' of hospitals in the Czech Republic.

In the current Czech system, hospitals guarantee quality and accessibility of health care for everyone and to serve that purpose they have to become financially stable and efficient. However, the majority of Czech public hospitals record financial losses [30] caused by a range of obligatory medical operations not covered by mandatory health care insurance. Figure 3 shows the breakdown of ownership of hospitals in the Czech Republic (data from [17]).

Table 2 presents a summary comparing the key features of the structure and functioning of each country's public hospital governance system. These are elaborated upon in the discussion section.

**Table 2:** Comparison of key features of public hospital governance

	Czech Republic	New Zealand
Governing bodies	State hospitals – no board of directors or supervisory board Municipal hospitals governed by board of directors. Members are hospital employees, municipal representatives and business people. Different number of members.	Public hospitals divided into 21 District Health Boards (DHBs), which serve as Boards of Directors for their hospitals. Some are hospital employees and some are self-employed or employed by other organisations 11 members per DHB
Membership of a governing body	State hospitals – no governing body. Municipality hospitals – members appointed by town and municipality officials.	7 members elected through public vote every 3 years, 4 are appointed by the Minister of Health (MoH). At least 2 members must be Maori.
Member's pay	Usually small fixed pay for a meeting. (Data not available).	Approx. US$15,000 annually.
Service delivery and financial targets	Vaguely set by the ministry, town or district.	Set by DHB Funding and Performance Directorate.
Accountability of governing body	Indirect.	Subject to 'public' control
Competence of a hospital director	High competence and high autonomy over both medical and financial results.	Hospital CEO has high status and is a top level executive appointment
Accountability of a director	Moderate financial involvement in potential profits. Threat of redundancy.	CEO is accountable to board for overall financial results and service delivery performance
Controlling body	State hospitals – ministries: low direct involvement, subject to political changes, unfocused. Municipality hospitals – town and district representations: low involvement, subject to political changes, unfocused.	DHB Funding and Performance Directorate and Ministry of Health, subject to political changes, focused.

## Discussion

In this section information from the preceding case study literature is discussed in relation to D W Taylor's [2] principles of 'good governance' which are outlined in Table 3. Each principle is considered from the New Zealand and then the Czech Republic perspective.

**Table 3:** D W Taylor's principles of 'good governance'

**Principle**	**Application**
1. Knowing what governance is.	CEO is responsible to board for implementing its policies plans and strategic directions. Board is responsible for developing corporate policies and pans; monitoring and measuring organisational performance against those policies and plans; and acting as a voice of the ownership of the hospital. Board's governance responsibilities are to provide a linkage between the hospital and its moral ownership; monitor the performance of the CEO; and develop an explicit statement of values for the hospital.
2. Achievement of strategic ends	To be effective by providing the right service, at the right place, at the right time, and at an affordable cost. Hospital governance structure must be such that performance objective can be set measured and accomplished.
3. Board-CEO relationship.	Relationship is typified by a high level of mutual confidence and trust throughout the organisation and particulalry between the board of directors and CEO. Governance viewed as a solemn partnership between board and CEO. Board members and the CEO are equals, colleagues. Organisations should be conceived of as a number of concentric circles with clients in the outermost circle and the CEO in the inner circle.
4. Unity of direction	The CEO and board should function as a common body to pursue a common end. There should be only one board of governance, one CEO, one strategic plan, mission or vision, at any one time.
5. Unity of command	Orders should be received from one superior only. Decision making authority should flow in a straight line from the top to the bottom of the organization.
6. Unity of accountability and responsibility	Authority is a derivative of responsibility. Every employee, including the CEO, must be held accountable for the exercise of authority in executing his/her responsibilities.
7. Ownership needs.	A hospital's board ultimate accountability is to the organisation's ownership.
8. Self-improvement and quality management	Continuous improvement should be part of an organisational philosophy and should permeate all hospital management and governance practice.
9. Understanding the cost of governance	These include; board member's personal opportunity costs, direct board meeting expenses, the costs of staff supporting boar activities, the costs associated with errors made by boards, and the costs of ineffectively structured governance-management-organisation relationships.

D W Taylor's [2] first principle of 'good governance' is to ***know what governance is***. In this regard, New Zealand DHBs have formal mission statements and clearly defined performance objectives, codes of conduct and procedures spelling out exactly what is required for the governance function. Each DHB is comprised of 11 directors; seven of whom are elected through a public election system for three years term, and four are appointed to positions by the MoH. The Chief Executive Officer (CEO) of the hospital is appointed separately by the board. Care is taken to ensure representation from all of the different community stakeholder groups (there is a legal requirement to appoint at least two Maori members to represent interests of New Zealand's indigenous people). The elected DHB members are legally required to establish consultation processes whereby providers are users of health care service, and the community has an opportunity to have an input into the major decisions made by the boards. Each board is subject to monitoring and an audit of both health and economic indicators of the hospitals, which is carried out by the DHB Funding and Performance Directorate [27].

While New Zealand public hospitals are governed by mixed-membership boards comprising medical staff, a CEO and representatives external to the organisation, Czech public hospital boards (if they exist) are appointed by authorities and typically consist of medical staff and local politicians. Governance in Czech hospitals is defined and determined by the owners' relationships with managers and there is a range of governing structures in different types of hospitals. A two-level governing board with a board of directors and a supervisory board comprising mostly owners is common [32]. Hospital managers are often appointed for a three to five year term by the municipality or the governmental authority according to their ownership. Due to the various ownership structures in the Czech Republic, governing principles are often inconsistent and they fail to emphasize the roles and activities which are supposed to be carried out by managers and owners. Some owners have not taken over their responsibilities seriously as they were 'gifted' hospitals during the transition process. In these cases owners have often been found to confuse their role with management tasks [33]. The recent transformation of the health care system and the resulting interactions among stakeholders has culminated in a situation where personal concerns often come before hospital performance [33,34]. A lack of efficient control mechanisms and strategic planning has also been recorded in the Czech hospitals [34]. This is not surprising however given the governing corporate structures in other Czech businesses are reported as being insufficient or less developed in general [35-38].

The second principle is the ***achievement of strategic ends (goals)***. Social and economic pressures are increasing on hospitals to be effective, i.e. to provide the right service at the right place with a high quality. The governance structure and organisational structure of a hospital must be such that both service and financial performance objectives can be met, measured and accomplished [2,39]. In New Zealand, hospitals are funded by the government on a population basis and DHBs are required to decide on priorities and objectives of financial resources allocation. DHBs are responsible for both funding and running acute care services in their jurisdictions and their financial activities are monitored by an external central government agency, the DHB Funding and Performance Directorate. This mitigates against misuse of funds, inappropriate use of power and creates pressure to operate in an economically efficient manner. During the inherent consultative processes, DHBs can re-allocate funds to areas with increased demand and reduce funds to areas where alternative options or services are no longer required. The community remains informed about and directly involved in decision making.

Although DHB have to maintain the financial stability of the hospitals, their major strategic goal is to improve, promote and protect the health of those within its district and to expedite the independence of people with disabilities within its district. Whilst not expected to return profit to government, hospitals are required to operate within pre-determined fixed budgets [4,27]. Boards are responsible for achieving non-financial goals such as promoting the health and independence of their populations. They are also required to assess the health and disability needs of the people in their regions and manage resources appropriately.

Principle three addresses the nature of the ***board-CEO relationship***. Boards have special responsibilities of providing a link between the hospital and its moral ownership, monitoring the performance of the CEO, managing the board-medical staff relationship, the board community relationship, intra-board relationships. The New Zealand style of relationship resembles Taylor's idea of the organisation being a series of stakeholders' concentric circles with the CEO in the inner circle. Although the board has ultimate accountability for decisions that are made, the CEO, clinical leaders and senior managers are all involved in top-level functions decision making which is done in consultation with the hospital's clients.

The nature of board-CEO relationships varies in the Czech Republic where privately owned Czech hospitals have top-down traditional pyramid ownership structures i.e. a two-level governing model applied in most of Central Europe [32], where a board of directors consists of company managers and a supervisory board represents owners and operate under standard profit oriented business principles. Hospitals owned by Czech municipalities (districts, towns) are governed by boards of directors consisting of between six and ten employees (mainly medical doctors), municipal representatives (not elected but appointed based on political concerns) and business professionals external to the organisation. This composition facilitates a close relationship between owners and managers. Interests of these three groups are usually quite distinct from each other and therefore corporate performance is not always regarded as the most important objective. Accordingly, disputes among the different in-groups result in higher autonomy of management at the expense of owners. Recently, some municipal public hospitals were transformed into publicly owned enterprises based on profit principles. It has been argued that controlling of overall performance, organisational structure and division of competencies would be better, but no substantial change is expected to happen [30].

The next three 'good governance' principles are derived from classical management principles which are interlinked. They concern the organisation and mechanistic functioning of boards. Principle four is ***unity of direction***, five is ***unity of command ***and six is ***unity of accountability and responsibility***. Collectively, these principles relate to the scalar principle of organisational design meaning that the chain of command should flow in a straight line from the top to the bottom of the organisation, i.e. from the board's CEO down through the various staff levels in the hospital, and that people in positions of power within the organisation should be accountable for their actions and directly responsible to their superiors.

In New Zealand, the DHB structure operates in a collegiate manner as it requires that the board consult with stakeholders before major decisions about the use of funds are made. Board meetings are formal and decisions are made by voting on issues after a period of discussion and debate. Nevertheless, final decisions made by boards are implemented in a top down fashion and individual board members are accountable to their community, CEO and also to the MoH. Hospital managers are also directly accountable to the board's CEO but this accountability is neither personal nor financial. However, in the Czech Republic, there is no similar clearly defined structure to direct board functions. Instead a director carries out some defined tasks but is not required to consult with stakeholders [30]. The lack of formal procedures and performance indicators is consistent with findings about generally poorer corporate governance in the Czech Republic [35]. Financial rewards for members of governing bodies are not dependent on hospital performance in either country and directors have a final decision making power without being personally liable for hospitals' performance.

Principle seven refers to ***ownership needs***. It points out that the board's ultimate accountability is to the organisation's owners, which in the case of public hospitals is the government. Even though New Zealand's DHBs are crown entities, whose boards are responsible to the MoH, only a minority of members (up to four) are appointed by the MoH. The majority of board members (seven) are elected by the community as their representatives. In regard to this board composition, it has been found that hospitals directed by mixed membership boards, i.e. including both members external and internal to the organisation perform financially better than hospitals whose governing bodies accommodate only people external to the organisation [40,41]. Furthermore, having a mixed representative structure mitigates against the appointment of people to positions because of political influence or favouritism which may happen in hospitals in the Czech Republic [31].

Principle eight embraces the notion of continuous ***self-improvement and quality management***. This principle is underpinned by the understanding that hospitals and health systems are not just economic but also social entities [40]. The New Zealand consultative mechanism and collegiate style of governance enables the public or social good function to be fulfilled the way that the DHB operates allows all members of the geographical area to participate in electing people to represent their views. Direct consultation with board members is possible and the members of the public are invited to attend all board meetings. The eighth principle has not yet been articulated in the Czech system.

The last principle relates to ***understanding the cost of governance ***and it addresses issues such as the payment of board members, direct costs of meetings, staff supporting board activities, costs associated with errors made by boards, etc. In regard to the issue of financial and opportunity cost of board members' time, DHB members are paid a reasonable amount for their work (NZ$23,000, i.e. approximately US$15,000 annually) but in the Czech Republic, board members (where they exist) are not paid in the same manner [41]. The DHBs are subject to regular audit process through the DHB Funding and Performance Directorate to ensure funded services are financially viable, clinically safe and of a high quality. Information about the operation of public hospitals is made available through government reports [4]. In the Czech Republic, hospital's annual financial results are not subject to unified control mechanisms, although some municipal and government authorities carry out regular checks. However, in regard to this point, it has been suggested that auditors should be selected externally and not appointed by health authorities [38], which is not applicable to either country.

## Conclusion

Financial, economical, or governance discussions about health care systems are perceived with some ambiguity and tension. It is believed that health care systems are notoriously difficult to manage and "almost everywhere reforms are being contemplated, organized, or implemented, some in direct contradiction to others. Each is claimed to make the system more responsive to user needs, yet most are really designed to bring its component parts under control – particularly financial control" [[42]:58].

From this literature review and comparative case analysis it is concluded that many of Taylor's [2] principles of 'good governance' are apparent in the current New Zealand health care system. These include *knowing what governance is, achievements of strategic goals, ownership needs, self improvement and quality management*, and *understanding the cost of governance*. New Zealand now has reached a period of stability and its' hospitals are administered by a system of elected boards, i.e., a collegiate style of governance where a group of paid individuals work together for a common purpose with supposedly equally shared authority [43] and this appears to fit with Taylor's [2] ideas about board-CEO relationships. Collegiate governance is closer to commercial activities in the form of partnerships which are the usual form of running small businesses and providing professional services [1,43]. One benefit of the collegiate system and mixed member composition of a board is that it provides a voice for public ownership and accountability. In comparison, the Czech system where board members are government appointees seems to be detached from community needs.

A drawback of the collegiate style of governance relates to the issue of leadership which may be difficult because of the large number of board members who are involved in decision making. Another problem with this governing style may arise where board members and the CEO are equal colleagues, and there is a possible conflict of interest when the CEO sits as a voting member [39]. These are issues that need special consideration in investigating the *board-CEO relationship *principle of good governance. The collegiate board structure might also make it difficult to practice some of the 'top-down' management principles, i.e. *unity of direction, unity of command *and *unity of accountability and responsibility *because of the more time consuming consultative and participative style of decision making.

The Czech health care governance is still developing. It seems to lack several structural elements that underpin Taylor's nine principles of 'good governance' such as formal appointment procedures or defined performance goals. The hospital governance style seems to be 'ad hoc', i.e. inconsistent styles of governing and board composition are used in public hospitals. However, several advantages may exist for Czech hospitals as a result of such a non uniform approach, e.g. boards that are smaller in number may be more ready for action and equally as effective in achieving strategic goals, even though the appointment of the 'right representative mix' of people cannot be guaranteed. Nonetheless, an associated concern about a system where people are appointed, is a chance that a CEO might be swayed by employees or political representatives holding positions of power inside the hospital management structure to make decisions favourable to themselves, rather than for the greater good of the public [44].

Additionally, while board members are paid a modest sum of money for their work in New Zealand, both governing systems do not fully address the issue of board members' financial *cost of governance*. This is signalled as an important issue for the future as recent research has found that in situations where board members and CEOs are underpaid, selection and performance problems are more likely to arise [45]. Adequate reimbursement for the highly skilled work of governing is therefore essential if quality services are to be provided in health care [46].

To conclude, this case study analysis has demonstrated that although the New Zealand and Czech Republic health systems appear to show a large degree of convergence, also evident is the fact that the resultant approaches to public hospital governance differ on several counts. This not surprising While some, but not all nine of Taylor's [2] principles of 'good governance' were found to exist in each country's hospital systems, the current styles of governance clearly differ. There are several possible implications for the theory of 'good governance' in health care. First, the 'set' of nine principles may need refinement based on further empirical research as they were not observed universally in this study. Second, D W Taylor's comment that the emergence of new governance models "defy the principles of good governance" [[2]: 108] may be unfounded because governance styles have evolved from particular historical circumstances and they appear to meet the requirements of different health care contexts. A third possibility is that the governance principles in general are best suited to the corporate world as they do not fully reflect the special nature of health care. For the future then, it might be necessary to ensure that the theoretical principles of 'good governance' are more strongly intertwined with the practical and social good requirements that are inherent in the delivery of hospital services.

## Competing interests

The author(s) declare that they have no competing interests.

## Authors' contributions

All authors participated in the conception and general idea of the article. PS compiled the first draft of the study, PP provided the context for Case 2, and ED introduced the context of Case 1. Further refinements were carried out mainly by ED with assistance of PS.
